# Amyloidosis Presenting with Macroglossia

**DOI:** 10.7759/cureus.3185

**Published:** 2018-08-22

**Authors:** Amir Shahbaz, Kashif Aziz, Muhammad Umair, Zohra R Malik, Saba Iqbal Awan, Issac Sachmechi

**Affiliations:** 1 Internal Medicine, Icahn School of Medicine at Mount Sinai/Queens Hospital Center, New York, USA; 2 Internal Medicine, Icahn School of Medicine at Mount Sinai Queens Hospital Center, New York, USA; 3 Internal Medicine, Icahn School of Medicine at Mount Sinai Queen Hospital Center, West Hempstead, USA; 4 Internal Medicine, Icahn School of Medicine at Mount Sinai/Queens Hospital Center, New York City, USA; 5 Diabetes Center, Ichan School of Medicine, New York City, USA

**Keywords:** amyloidosis, macroglossia

## Abstract

Macroglossia is an uncommon condition that causes cosmetic and functional disabilities. We present a case of a 67-year-old patient with the past medical history of vitamin B12 deficiencies who presented with macroglossia and was found to have amyloidosis. She had an enlarged tongue with multiple ulcerations secondary to traumatic injury from dentation along with difficulty swallowing. Laboratory workup was unremarkable apart from elevated C reactive protein (CRP) and low complement 3 (C3) levels. On the second day of admission she had gastrointestinal bleed; computed tomography (CT) scan of the abdomen with oral contrast was performed which revealed nodular thickening of the stomach suspicious for gastric malignancy. Endoscopy was postponed as there was concern that macroglossia could comprise the airway. A biopsy of the tongue was performed and histological staining was positive suggestive of the amyloidosis. We discuss here the probable underlying causes of macroglossia and amyloidosis.

## Introduction

Macroglossia is an abnormal enlargement of the tongue [1]. It is an uncommon condition that causes cosmetic and functional disabilities in speaking, eating, swallowing, and sleeping. Oral amyloidosis is a rare and debilitating condition that, whether primary or secondary, may severely impact the quality of the patient’s life. The differential diagnosis includes hypothyroidism, growth disorders, neoplasm, chromosomal abnormalities, and amyloidosis. Vitamin deficiencies, particularly Vitamin B12, cause B12 deficiency glossitis (inflammation of the tongue) mimicking macroglossia [1]. We reported a case of a patient with the past medical history of vitamin B12 deficiency who presented with macroglossia and was found to have amyloidosis.

## Case presentation

We presented a case of a 67-year-old female nursing home resident with a history of diabetes mellitus type 2, hypertension, hyperlipidemia, old ischemic stroke, bronchial asthma, and pernicious anemia on monthly vitamin B12 injection. She was admitted to our hospital with complaints of persistent diffuse joint pain and several tongue ulcerations secondary to traumatic pressure from dentation and associated difficulty swallowing. Otorhinolaryngology and rheumatology services evaluated her and treated her with a course of amoxicillin-clavulanate, acyclovir for 14 days as well as with a short course of oral steroids showing improvement. The serology for herpes simplex was negative. Rheumatological workup was unremarkable apart from elevated C reactive protein (CRP) and low complement 3 (C3) level. She was discharged to a nursing home with a plan to follow-up as an outpatient, but no follow-up was recorded. Two months later she returned with complaints of generalized joint pain and poor intake due to difficulty swallowing. Review of her medical record revealed dysplastic changes during endoscopy in March 2009 showing gastritis and peptic duodenitis. Computed tomography (CT) chest in April 2009 showed incidental left lung nodule and was unchanged on repeat CT chest in October 2009. On physical examination, her blood pressure was 133/70 mmHg, heart rate was 89/min, and oxygen saturation on room air was 99%. She had an enlarged tongue, swollen and tender with several ulcerations between 0.1 and 1 cm in size and with a white discharge. She also had submandibular nontender lymphadenopathy and bilateral joint swelling of shoulder, knee wrist, and elbow. She had normocytic anemia at baseline, mildly elevated white blood cell count 11,000/ml (reference range: 4000-10,000/ml), and elevated blood urea nitrogen/creatinine from a normal baseline two months ago. The urinalysis was negative for protein and positive for trace blood. On the second day of admission, she had gastrointestinal bleed with a drop of her hemoglobin from 8 to 6.4 mg/dl. The CT abdomen with an oral contrast was performed which revealed nodular thickening of the stomach suspicious for malignancy and pelvic ascites with high-density fluid. Endoscopy was postponed as there was concern that macroglossia could comprise the airway.

Further workup did not show any spikes on serum protein electrophoresis. Antinuclear antibody (ANA) and rheumatoid factor were negative. Complement 4 (C4) was normal and repeat complement 3 (C3) was low. Thyroid function test, C1 esterase inhibitor, and insulin-like growth factor 1 (IGF-1) were normal. Renal function test improved subsequently but did not return to baseline. Ultrasound of kidney showed changes suggestive of the renal parenchymal disease. We started prednisone 15 mg twice daily as per rheumatology consultation and observed marked improvement in her joint pain. The tongue remains unchanged without further discharge. A biopsy of the tongue was performed and histological examination was suggestive of amyloidosis.

## Discussion

Extracellular deposition of highly insoluble fibrillar proteins caused amyloidosis. Virchow adopted the term “amyloid,” meaning starch or cellulose, to describe abnormal extracellular proteins. Different proteins are known to produce amyloid fibrils in human, most of them being constituents of plasma. The normally soluble precursor proteins, by the substitution of amino acids, precipitate when provoked by physical or chemical stimuli, such as the local surface pH, electric field, and hydration forces on cellular surfaces and get misfolded to form a beta-pleated sheet structure and get deposited as amyloid [2].

In primary systemic amyloidosis, the amyloid derived from monoclonal immunoglobulin light chain is called AL amyloid. Secondary amyloidosis was associated with many chronic inflammatory diseases and malignancies. Amyloid fibrils were derived from cleavage fragment of the circulating acute phase reactant serum amyloid A (SAA) protein, hence called as AA amyloid. In localized cutaneous amyloidosis, amyloid derived from keratin was released from apoptotic keratinocytes [3]. All amyloid proteins share common features including an amorphous eosinophilic appearance on light microscopy in H and E staining; bright green fluorescence was observed under polarized light after Congo red staining and beta-pleated structure using X-ray crystallography. Deposition of amyloid in tissues leads to distortion of tissue architecture, organ enlargement (organomegaly), and organ dysfunction [4]. Amyloidosis (AL) is known for a highly variable clinical presentation. Amyloid deposition leads to hepatomegaly (50%), splenomegaly (10%), peripheral neuropathy, renal amyloidosis, and carpal tunnel syndrome (25%). Cardiac involvement causes conduction defects, arrhythmias, congestive cardiac failure and may account for 40% of deaths [1].

Macroglossia is the most frequent oral manifestation of amyloidosis and may be found as the only presenting symptom of the disease. Before considering the presence of amyloid protein, other likely causes of tongue enlargement, such as malignant tumors of the tongue, vascular abnormalities, hypothyroidism and deficiency of vitamin B12 and folic acid, should be ruled out [1]. Macroglossia in our patient was secondary to amyloidosis and was confirmed by histological examination. Normal thyroid function tests make this unlikely secondary to hypothyroidism. Tongue biopsy did not reveal any local malignancy. Vitamin B12 deficiency may cause glossitis that mimics macroglossia. Our patient was taking vitamin B12 replacement therapy, and her B12 levels were normal. Our patient did not have cardiac involvement as indicated by normal ECG and chest X-ray. Arthropathy in our patient may be secondary to amyloid deposition in joints and other structures. She had a minimal renal parenchymal disease; although renal function tests returned to baseline the possibility of renal amyloidosis cannot be completely ruled out. Renal involvement presents with proteinuria and renal failure. It is one of the bad prognostic indicators and indicated by proteinuria and USG renal parenchymal changes [1].

The primary systemic amyloidosis is a diagnosis of exclusion. Our patient may have either primary or secondary amyloidosis. As serum protein electrophoresis did not show any spike, myeloma-associated amyloidosis was ruled out. The patient record revealed dysplastic changes in previous endoscopies and CT with contrast showed revealed nodular thickening of the stomach suspicious for gastric malignancy. The secondary amyloidosis due to gastric malignancy may likely explain the overall presentation of our patient. Alternatively, gastric bleeding may be secondary to gastric amyloidosis [5-7]. Radiographically, gastric amyloidosis causes diffuse thickening of the mucosal folds. This finding carries a broad range of differential diagnosis [8]. The patient will await endoscopy and biopsy for the final conclusion.

Treatment of amyloidosis aimed at reducing the supply of precursor proteins, preventing the disability and organ damage. Prognosis in AL amyloidosis is poor. The main causes of death in AL amyloidosis are the cardiac and renal failure. In AL amyloidosis, cardiac and renal failure are the major causes of death. The median survival of patients with myeloma-associated amyloidosis is five months and 2.1 years for patients with primary systemic amyloidosis [9]. Conservative excision is a satisfactory treatment for local amyloid masses; the role of surgery in systemic forms is controversial. Partial glossectomy via a pull-through approach has been utilized in select cases [10]. There are no specific guidelines to aid clinicians with the management of macroglossia secondary to amyloidosis although numerous therapies have been proposed, including surgical excision and pharmacological treatment. The prognosis is uncertain, owing to the rarity of the condition, requiring regular follow-up and monitoring [11].

## Conclusions

A high index of suspicion is necessary for the diagnosis of such rare cases. In a patient presenting with macroglossia and tongue ulceration, one should not dismiss the possibility of amyloidosis.
